# Green biomimetic synthesis of Ag–TiO_2_ nanocomposite using *Origanum majorana* leaf extract under sonication and their biological activities

**DOI:** 10.1186/s40643-020-00357-z

**Published:** 2021-01-02

**Authors:** Diksha Bhardwaj, Ruby Singh

**Affiliations:** Department of Chemistry, School of Basic Sciences, Jaipur National University, 302017 Jaipur, Rajasthan India

**Keywords:** *Origanum majorana*, Antimicrobial, Antioxidant, Ag–TiO_2_ NCs

## Abstract

**Background:**

Studies of plant extract-mediated synthesis of nanoparticles is extensively explored and studied in recent time due to eco-friendly, cost-effectiveness and minimal use of toxic chemicals for synthesis. In this study, the synthesis of Ag–TiO_2_ nanocomposites (NCs) was carried out using *Origanum majorana* leaf extract under ultrasound irradiation. *Origanum majorana* leaf extract plays an important role as reducing and capping agent in synthesis of Ag–TiO_2_ nanocomposites (NCs). The antimicrobial activities of synthesised Ag–TiO_2_ NCs have been studied against Gram-positive and Gram-negative bacteria. In addition to this, the antioxidant activity of green Ag–TiO_2_ NCs was also evaluated on the basis of free radical scavenging activity against 1,1-diphenyl-2-picrylhydrazyl (DPPH), 2,2′-azino-bis(3-ethylbenzthiazoline-6-sulfonic acid (ABTS), and hydrogen peroxide free radicals.

**Results:**

Green-synthesised Ag–TiO_2_ NCs were successfully characterised on the basis of UV–Vis spectrophotometer, Fourier transform infrared (FT-IR) spectroscopy, X-ray diffraction analysis (XRD), scanning electron microscopy energy-dispersive X-ray spectroscopy (SEM–EDS) and transmission electron microscopy (TEM). The results revealed the spherical shape of nanocomposite with an average size 25–50 nm. The synthesised Ag–TiO_2_ NCs have showed significant antimicrobial activity against *Escherichia coli*, *Bacillus subtilis* and *Aspergillus niger* in comparison to TiO_2_ nanoparticles (NPs). The antioxidant evaluation of biomimetic synthesised Ag–TiO_2_ NCs also exhibited strong activity than TiO_2_ NPs and comparable to standard.

**Conclusion:**

Green-synthesized Ag–TiO_2_ NCs provide a promising approach that can satisfy the requirement of large-scale industrial production bearing the advantage of low cost, eco-friendly and reproducible.
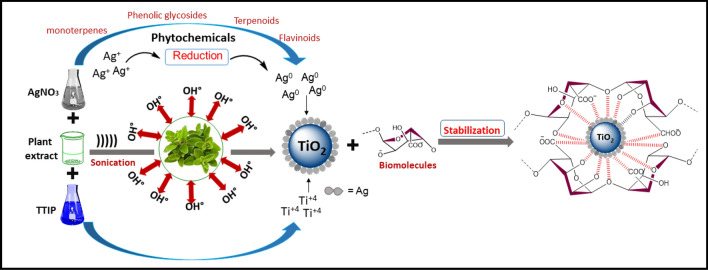

## Background

Nanotechnology is an emerging as a rapidly growing field with its applications in science and technology and nanostructures are important tool in different areas of research (Manke et al. 2013; Hussain et al. 2016; Ostovan et al 2017). Metal and metal oxide nanoparticles (NPs) have received considerable research attention due to their exceptional electrical, optical, magnetic, catalytic and pharmacological properties. Traditional methods for the synthesis of metal and metal oxide NPs include reducing and stabilising chemical agents that are expensive and have an adverse effect on the environment (Guo et al. 2015). In response, researchers are now looking for alternative “green synthesis” approaches in an effort to reduce or eliminate harmful chemicals during the production of NPs (Singh et al. 2018). Green synthesis of metal and metal oxides using plant extract has been extensively studied in recent time in eco-friendly manner with using minimum amount of hazardous chemicals (Zheng et al. 2015; Yulizar et al. 2018, 2020, Yulizar and Apriandanu 2019; Pirtarighat et al. 2019).

Among various metal oxides, titanium dioxide (TiO_2_ NPs) have demonstrated as a most valuable material in various fields due to its unique surface chemistry, high chemical stability, non-toxicity and clean photocatalytic nature with great morphologies that have significant impact on both academia and industries especially in biomedicine field (Wu 2011; Daghrir et al. 2013; Shi et al. 2013). TiO_2_ NPs are environmentally harmonious material having remarkable biological activities including antibacterial (Visai et al. 2011), antioxidant (Sethy et al. 2020), anti-parasitic (Durairaj et al. 2014) and anticancer activities (Trouiller et al. 2009). The two inherent properties of such as large bandgap and the fast recombination of electron–hole pairs are make the applicability of TiO_2_ limited (Chai et al. 2017; Monfort et al. 2017).

Recent developments in the synthesis of nanomaterials with metal nanoparticles (Ag, Au, Fe, Cu, Ru, and Pd) deposited on metallic oxide surfaces have gained considerable curiosity in nanotechnology and material science because of their significant applications in diverse fields such as biomedical, catalysis, biosensing, information storage, solar cells, optical and many more (Liu et al. 2011; Gawande et al. 2016; Zheng et al. 2016; Zhang et al. 2018; Yulizar et al. 2019). Silver and gold nanoparticles have been increasingly used due to their powerful optical, electrical and microbial properties in various areas of research as biological sensors, catalysis, drug delivery vehicles, and antimicrobial agents as well as having low cytotoxic effects on mammalian cells (Mahmoudi and Serpooshan 2012; Crites et al. 2013; Padmos et al. 2015).

Moreover, investigations reveals that Ag-doping regulates the cytotoxicity of TiO_2_ NPs by selectively kill human cancer cells while sparing normal cells (Ahamed et al. 2017). Recently, it was explored that without any light illumination, Ag–TiO_2_ produces a typical ROS (reactive oxygen species) potential in killing microbial communities (Lin et al. 2011). Besides this, generation of ROS also causes damage to protein, lipids and rupture the DNA cells which confirms Ag–TiO_2_ NCs as potential antibacterial agent (Jin et al. 2011). Thus, Ag–TiO_2_ NCs are a suitable candidate due to its simple and inexpensive synthesis, high availability, low-toxicity with unique optical-physio and biological properties.

Synthesis of Ag–TiO_2_ NCs has been reported by various methods such as sol–gel process (Zhao and Chen 2011), chemical vapour deposition (Lee et al. 2014), thermal dissociation (Saravanan et al. 2018) and electrochemical oxidation (Avciata et al. 2016). Most of these physio-chemical methods not only violates green principles but also suffers irreversible agglomeration and poor dispersibility of nanoparticles causing weak biological activities. Hence, to fulfil these limitations, nowadays the focus of researchers have shifted towards the plant extract-mediated synthesis of diverse nanomaterials but little attention has been paid on the synthesis of Ag–TiO_2_ NCs using plant extracts. Recently, green synthesis of Ag–TiO_2_ NCs using different plant extracts has been reported such as *Citrus lemon* (Liang et al. 2012), *Acacia nilotica* (Rao et al. 2019), *Nephelium lappaceum* (Kumar et al. 2016) and *Euphorbia heterophylla* (Atarod et al. 2016) for improved photocatalytic and biological activities. However, these synthesis favours green methodology, but in most of cases, synthesis of NCs completed in two or more steps and conventionally requires much time for reaction process. In this regard, the use of ultrasound as a non-conventional energy source is an effective alternative eco-compatible approach in materials synthesis (Xu et al. 2013). Ultrasounds in nanomaterial synthesis reduced the time and some reports proved the formation of lower particles size due to its cavitational effect (Neppolian et al. 2008; Jordens et al. 2016). During cavitation, bubbles are formed and subsequently collapse to form non-aggregated nanoparticles in short reaction time with high yields.

Keeping in view the diverse applications and limited literature on plant-mediated synthesis of Ag–TiO_2_ NCs, in the present study, we have reported a green *Origanum majorana* leaf extract-mediated synthesis of Ag–TiO_2_ NCs under sonication for the first time. The leaves of medicinally important plant *Origanum majorana* (lamiaceae) contain flavonoids, phenolic terpenoids, phenolic glycosides and oxygenated monoterpene (Goel and Vasudeva 2015), and these biomolecules act as the reducing and stabilising agents in the nanomaterial synthesis (Singh et al. 2016; Mohammadian et al. 2018; Nezhad et al. 2020).

## Materials and methods

Leaves of *Origanum majorana* were collected from the local area of Jaipur district, Rajasthan, India. The chemicals used in the present report were Titanium (IV) isopropoxide [Ti{OCH(CH_3_)_2_}_4_], silver nitrate (AgNO_3_), 1,1-diphenyl-2-picrylhydrazyl (DPPH), 2,2′-azino-bis(3-ethylbenzthiazoline-6-sulfonic acid (ABTS), hydrogen peroxide, butylated hydroxyltoluene (BHT) and ascorbic acid. All the chemicals used in the experiments were purchased from Merck Chemical Company (Darmstadt, Germany) and used as such without further purification.

### Characterisation methods and instruments

Green-synthesised Ag–TiO_2_ NCs were characterised using UV–Vis, FTIR, XRD, SEM–EDS and TEM analysis. Perkin-Elmer LAMBDA 750 UV–Vis NIR spectrophotometer was used taking quartz cuvette with de-ionised water as a reference. FTIR spectra were recorded on a Perkin-Elmer spectrum version 10.4.00 and using a spectral range of 4000–400 cm^−1^ with KBr pellets. XRD measurements were carried out on X-ray diffractometer (Panalytical X Pert Pro) equipped with a CuKα radiation (*λ* = 1.54060 Å) operated at a voltage of 45 kV and current 40 mA. Surface morphology and shape of the NCs were estimated by SEM [Nova Nano FE-SEM 450 (FEI)]. The FE-SEM is coupled to EDS detector for identification of elements present in nanoparticles and to analyse its chemical composition. Transmission electron microscope was recorded using a Tecnai G2 20 (FEI) S-Twin 200 kV. The ultrasound-assisted reactions were carried out using an ultrasonic processor probe system (Qsonica700) operating at 20 kHz, 700 W with 12 mm tip. The operating conditions were a 30-s pulse on and 30-s pulse off time with an amplitude of 50% for 10 min.

### Preparation of *Origanum majorana* leaf extract

The collected leaves of *Origanum majorana* were washed thoroughly under running tap water to remove the associated dust particles and dried. For preparation of leaf extract, about 20 g of dried leaves of *Origanum majorana* was powdered and then 100-ml deionized water was added and heated upto 80 °C for 10 min. The mixture was further allowed to cool down to room temperature, then the mixture was filtered using Whatman no.1 filter paper. The residue was removed and filtrate was used for the synthesis of NCs.

### Synthesis of Ag–TiO_2_ NCs using ***Origanum majorana*** leaf extract

For green synthesis of Ag–TiO_2_ NCs, precursor solution 0.1 M of titanium (IV) isopropoxide (TTIP) with 15-ml deionised water was placed in a cylindrical glass vessel and irradiate for 10 min under sonication. After that, 1 mM AgNO_3_ solution with 25-ml leaf extract of *Origanum majorana* was added dropwise with continuous sonication. After 10 min, the colour of the solution changed to grey due to the excitation of surface plasmon resonance (SPR) which indicated the formation of Ag–TiO_2_ NCs. The resultant NCs solution was then centrifuged at 6000 rpm for 20 min to complete the precipitation process of Ag–TiO_2_ NCs. The obtained precipitate was then washed three times with water to remove by-products and then dried at 80 °C in the oven for overnight followed by annealing at 400 °C for 2 h.

### Antimicrobial activities

The antimicrobial activity of synthesised Ag–TiO_2_ NCs was studied by standard Agar Well diffusion method as reported previously (Nguyen et al. 2019). The antibacterial activity was carried out against both Gram-positive (*Bacillus subtilis*, *Staphylococcus aureus*) and Gram-negative (*Escherichia coli*, *Pseudomonas aeruginosa*) pathogenic microorganisms and antifungal activity was evaluated against two selected fungi *Aspergillus niger* and *Aspergillus solani*. Each strain was swabbed uniformly onto the sterile nutrient agar Petri plates using cotton swabs. Wells of 8 mm diameter were then punched in the inoculated plates using a sterile plastic rod. Using a micropipette, four concentrations (25, 50, 75 and 100 µg/ml) of synthesised Ag–TiO_2_ NCs were loaded to the labelled wells, respectively. After incubation at 37 °C for 1 day, the diameter of inhibition zone was measured in millimetres (mm) using standard scale to determine the antimicrobial activity. The results of antimicrobial activity of synthesised Ag–TiO_2_ NCs were also compared with previously synthesized green TiO_2_ NPs using *Origanum majorana* leaf extract (Bhardwaj et al. 2019) to estimate the efficiency of Ag loading in NCs. The experiment also included reference standard.

### Antioxidant activity

Antioxidant activity of the green synthesised Ag–TiO_2_ NCs and TiO_2_ NPs were determined and compared with standard on the basis of DPPH, ABTS and hydrogen peroxide-scavenging assays.

#### DPPH free radical-scavenging assay

The antioxidant activity of green-synthesised Ag–TiO_2_ NCs and TiO_2_ NPs were investigated on basis of DPPH as described by earlier method (Miliauskas et al. 2004). Various concentrations of synthesised Ag–TiO_2_ NCs and TiO_2_ NPs were prepared and added to 1 ml DPPH solution (0.1 mM DPPH in methanol) in the test tubes labelled accordingly. The reaction mixture was shaken and then incubated for 30 min in dark place at room temperature. The absorbance was recorded spectrophotometrically at 517 nm. BHT was used as the reference standard antioxidant compound.

#### ABTS radical-scavenging assay

ABTS free radical-scavenging activity of green-synthesised Ag–TiO_2_ NCs and TiO_2_ NPs were analysed according to the reported method described earlier with moderate modifications (Li et al. 2011). The stock solution of ABTS radical cation (ABTS^+.^) was prepared by reacting 7 mM of ABTS stock solution with 2.45 mM potassium persulfate (K_2_S_2_O_8_). After incubation at room temperature for overnight, the absorbance was recorded at 734 nm. Synthesised Ag–TiO_2_ NCs and TiO_2_ NPs were separately added with ABTS at different concentrations and again incubated for 15 min in the dark place. ABTS reagent without sample was used as control solution.

#### Hydrogen peroxide-scavenging assay

The hydrogen peroxide-scavenging potential of green-synthesised Ag–TiO_2_ NCs and TiO_2_ NPs were determined according to reported method (Bhakya et al. 2016). A hydrogen peroxide solution was prepared in phosphate buffer at pH 7.4. The different concentrations of Ag–TiO_2_ NCs and TiO_2_ NPs with ascorbic acid (reference) were taken in test tubes and mixed with 50 μL of 5 mM hydrogen peroxide solution. Afterwards, the mixture was incubated for 10 min at room temperature and absorbance was measured spectrophotometrically at 230 nm against a blank solution containing phosphate buffer without hydrogen peroxide. The percentage of H_2_O_2_-scavenging activity was calculated using a control (blank) and sample (nanocomposites treated) absorbance.

## Results and discussion

### UV–Vis spectrophotometer

**Fig. 1:**
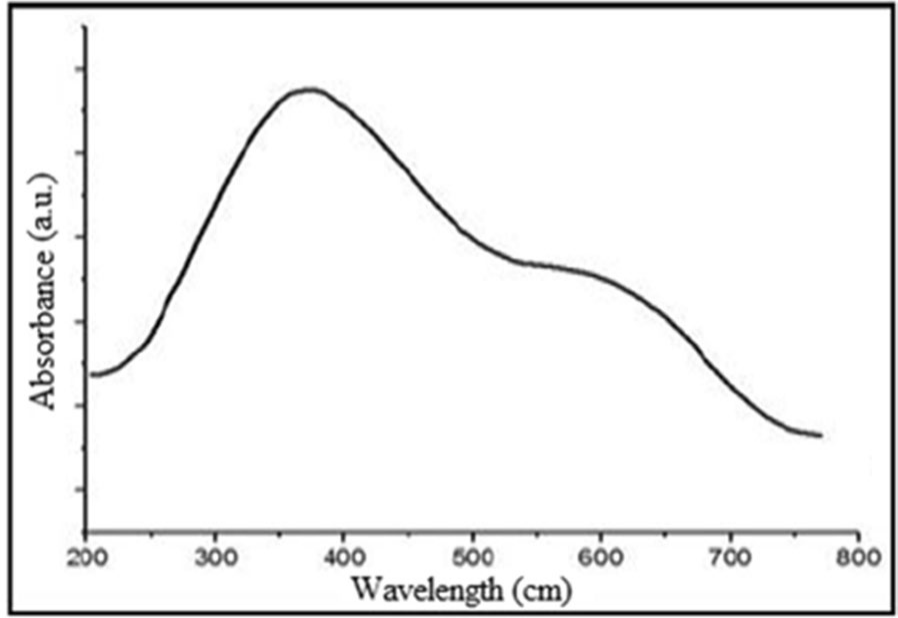
UV–Vis absorption spectrum for Ag–TiO_2_ NCs

UV–Vis spectrum of Ag–TiO_2_ NCs is presented in Fig. 1. The absorption band observed at 380 nm is due to TiO_2_ (Tang et al. 2012) and absorption at around 570 nm is due to localised surface plasmon resonance (LSPRs) of deposit Ag NPs on the surface of NCs (Zheng et al. 2011; Ramchiary et al. 2014).

### FTIR analysis

**Fig. 2:**
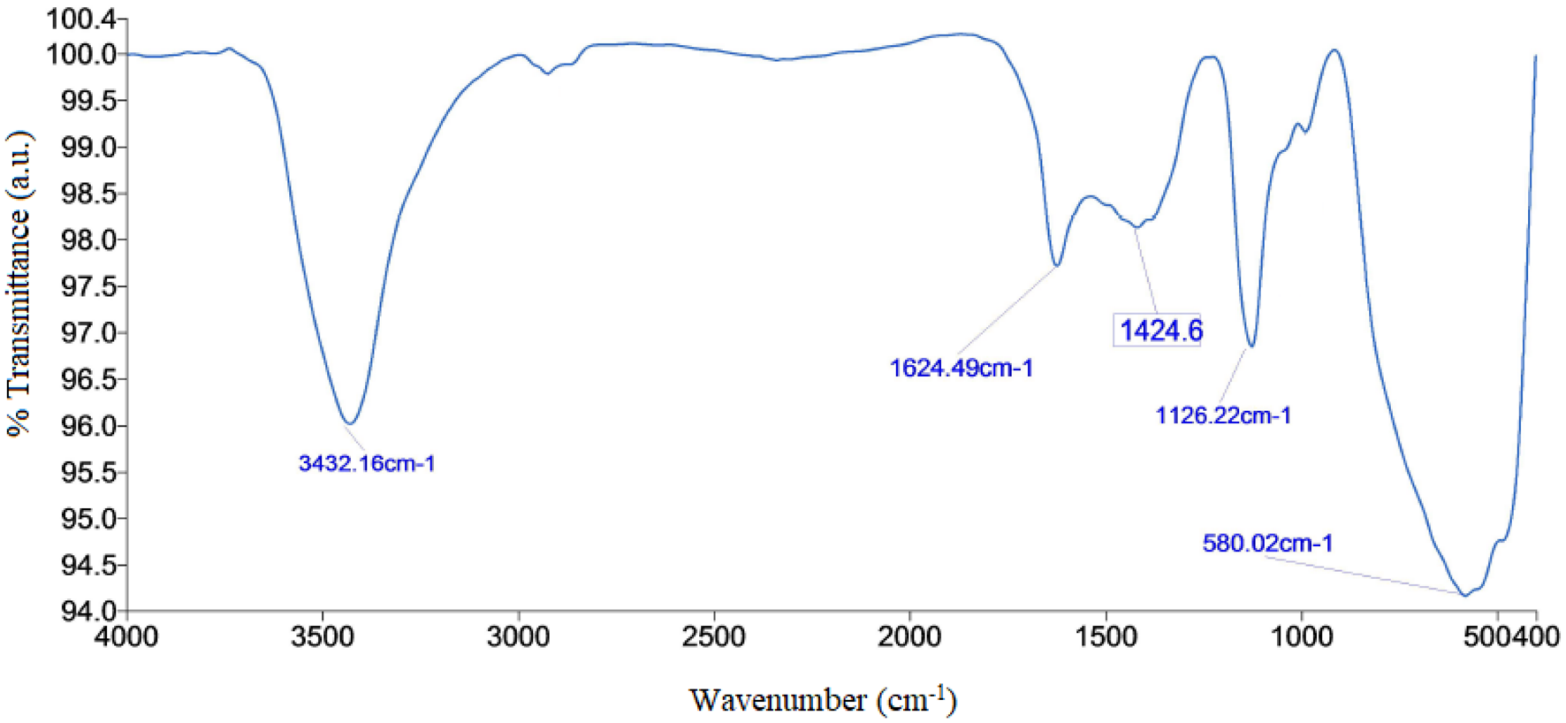
FTIR spectrum for green-synthesised Ag–TiO_2_ NCs

FTIR spectrum of Ag–TiO_2_ NCs (Fig. 2) shows characteristics peaks of OH groups corresponding to water molecules absorbed on the surface of the nanoparticles. Broad peaks at 3432 cm^−1^ and small peaks at 1624 cm^−1^ can be attributed to the stretching and bending vibration modes of water molecule, respectively (Llano et al. 2014). The strong absorption band at 400–600 cm^−1^ is attributed to Ti–O vibration band. Sharp peak around 550–760 cm^−1^ found in FTIR spectrum can be attributed to Ti–O–Ti bonding (Fleaca et al. 2015).

### XRD

**Fig. 3:**
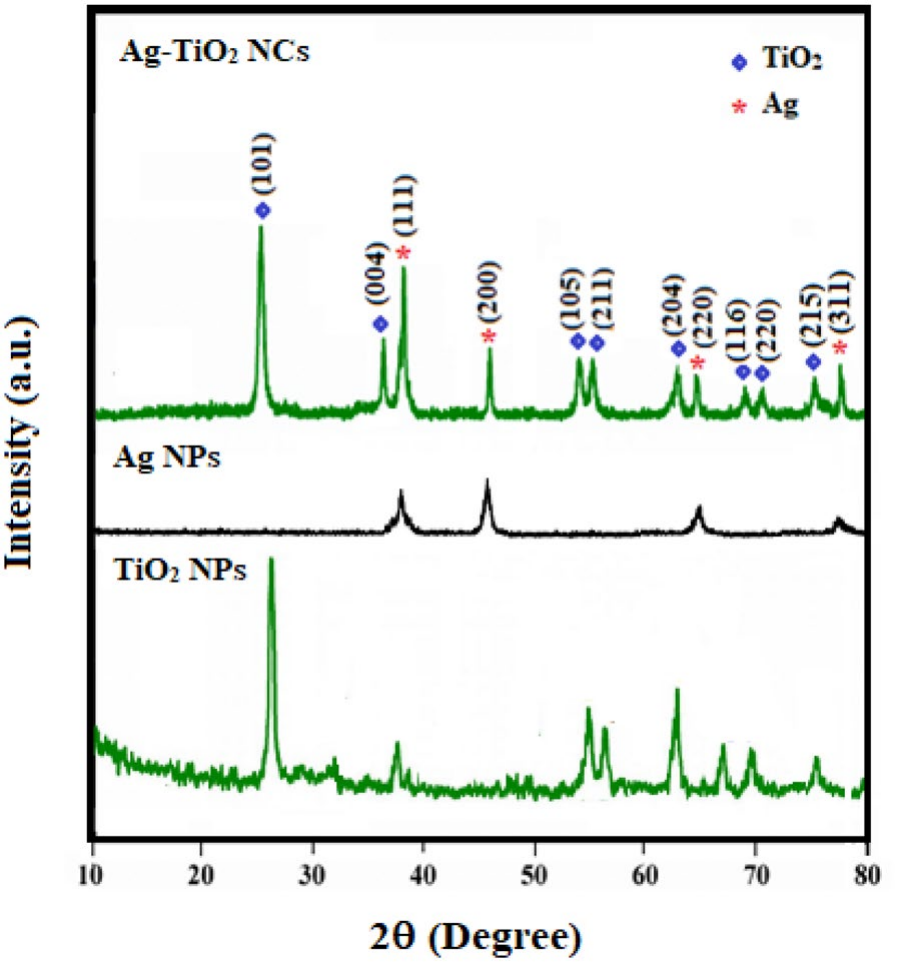
XRD patterns of Ag–TiO_2_ NCs, TiO_2_ NPs and Ag NPs

X-ray diffraction measurements are used for phase investigation and crystallinity of the nanomaterials. Figure 3 illustrates the XRD pattern of Ag–TiO_2_ NCs, bare TiO_2_ NPs and Ag NPs. The diffraction peaks in a wide range of 2θ angle are at about 28.25°, 36.80°, 44.05°, 54.89°, 56.06°, 64.69° and 69.96° corresponds to the crystal planes of (101), (004), (200), (105), (211), (204) and (116), respectively, attributed to the formation of tetragonal anatase phase of TiO_2_ nanoparticles (Chaiyo et al. 2017) (JCPDS card no. 01‐075‐2550). For Ag NPs, the main characteristic peaks at 2*θ* values are 38.11°, 44.27°, 64.42° and 77.47° which belonged to the (111), (200), (220) and (311) in lattice planes of face-centred cubic (FCC) structure approving the formation of Ag NPs (Yuan et al. 2010). The XRD pattern of Ag–TiO_2_ confirms the formation of dual phases including the anatase phase of TiO_2_ and the FCC lattice of Ag and indicates Ag particles did not enter into the crystal lattice of TiO_2_ and deposits only on its surface.

### SEM and EDS analysis

**Fig. 4:**
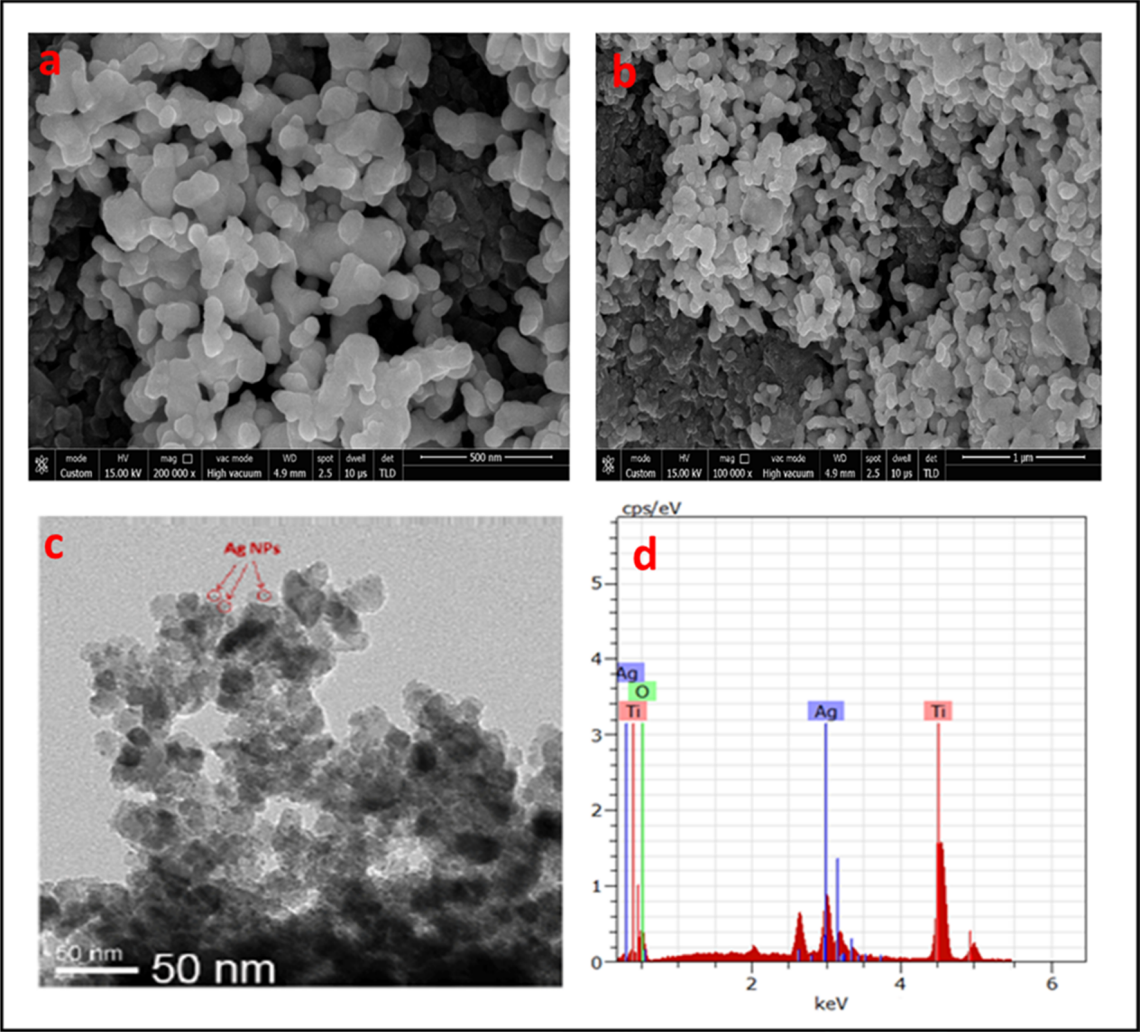
**a** SEM image of Ag–TiO_2_ NCs with magnification at 500 nm; **b** SEM image of Ag–TiO_2_ NCs with magnification at 1 µm; **c** TEM image of Ag–TiO_2_ NCs; **d** EDS spectrum of green-synthesised Ag–TiO_2_ NCs

The detailed morphology, particle size and shape of biomorphic Ag–TiO_2_ NCs was investigated by SEM and TEM analysis which showed the distribution of spherical shaped Ag particles on the surface of TiO_2_ which is not uniform. The average size of Ag–TiO_2_ NCs is 25–50 nm. The elemental composition of the Ag–TiO_2_ NCs was determined by EDS analysis and presence of titanium (Ti), silver (Ag) and oxygen (O) was confirmed (Fig. 4a–d).

### Plausible mechanism for the formation of Ag–TiO_2_ NCs

**Fig. 5:**
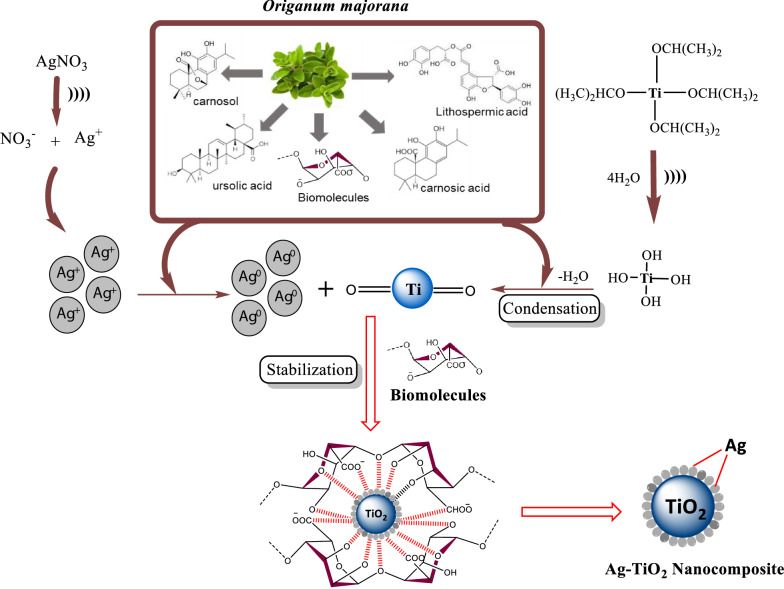
Plausible mechanism for the formation of Ag–TiO_2_ NCs using *Origanum majorana* leaf extract

The plausible mechanism for formation of Ag–TiO_2_ NCs is presented in Fig. 5. TTIP hydrolyse to Ti(OH)_4_ in aqueous media and Ti(OH)_4_ is usually not stable and hence, it would go through the condensation process to produce amorphous hydrous oxide precipitates (TiO_2_xH_2_O) as stated in the following equations (Mahshid et al. 2007):

1 $$ {\text{Ti}}({\text{OR}})_{4} + 4{\text{H}}_{2} {\text{O}}\mathop{\longrightarrow}\limits^{{({\text{hydrolysis}})}}{\text{Ti}}({\text{OH}})_{4} + 4{\text{ROH,}} $$

2 $$ {\text{Ti}}({\text{OH}})_{4} \mathop{\longrightarrow}\limits^{{({\text{condensation}})}}{\text{TiO}}_{2} {\text{xH}}_{2} {\text{O}} + (2 - {\text{x}}){\text{H}}_{2} {\text{O}}{.} $$

The presence of various hydroxyl groups in leaf extract of *Origanum majorana* were responsible for the antioxidant capacity and catalysed the condensation reactions (Roopan et al. 2012).

Simultaneously, AgNO_3_ separated to Ag^+^ and ${\text{NO}}_{3}^{ - }$ ions quickly in the aqueous solution as shown in the following equation:

3 $$ {\text{AgNO}}_{3} \mathop{\longrightarrow}\limits^{{{\text{Hydrolysis}}}}{\text{Ag}}^{ + } + {\text{NO}}_{3}^{ - } . $$

Consequently, reducing phytochemicals bind and capped the Ag^+^ ion to form the stable nanoparticles as presented in Fig. 5. The organic molecules which played the main role for formation of Ag–TiO_2_ NCs are carnosol, ursolic acid, carsonic acid, lithospermic acid and biomolecules present in the leaf extract of *Origanum majorana* (Bina et al. 2017).

Finally, formed Ag–TiO_2_ NCs were subjected to the calcination process at 500 °C to remove the water molecules.

### Antimicrobial activities of green-synthesised Ag–TiO_2_ NCs

**Fig. 6:**
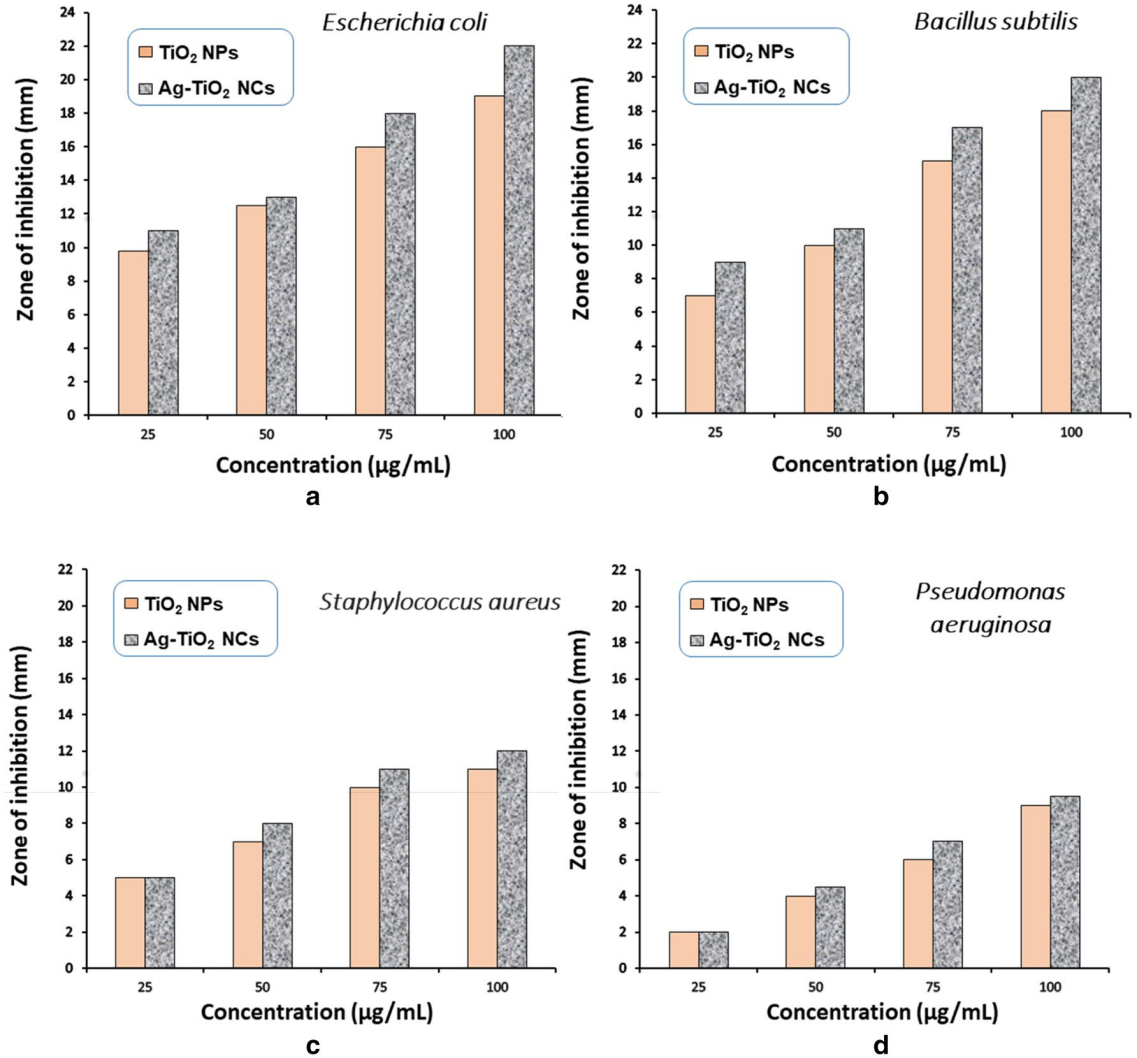
Antibacterial activity of green-synthesised Ag–TiO_2_ NCs and TiO_2_ NPs at different concentrations against four pathogens **a** *E. coli*, **b** *B. subtilis*, **c** *S. aureus* and **d** *P. aeruginosa*

The antibacterial assay of green-synthesised Ag–TiO_2_ NCs and TiO_2_ NPs were evaluated against pathogens of both Gram-positive *Staphylococcus aureus* and *Bacillus subtilis* and Gram-negative *Escherichia coli* and *Pseudomonas aeruginosa* bacteria. Figure 6 shows the zone of inhibition was observed on bacteria due to the effect of synthesised Ag–TiO_2_ NCs at four different concentrations compared with TiO_2_ NPs. The maximum zone of inhibition was noticed in *E. coli* (22 mm) followed by *B. subtilis* (20 mm) at 100 µg/ml concentration of Ag–TiO_2_ NCs. The lowest inhibition zone was observed with *P. aeruginosa* (2 mm) at 25 µg/ml concentration of Ag–TiO_2_ NCs. By doing experiments on different concentration of NCs, we found that zone of inhibition increases with the increasing concentration of Ag–TiO_2_ NCs (Table 1). This dose-dependent inhibition might be due to the denaturation of bacterial cell wall as NPs binds to cell membrane and pierced inside the bacteria followed by depletion of intracellular ATP (Adenosine triphosphate) (Mamonova et al. 2015). Synthesised Ag–TiO_2_ NCs exhibit good antibacterial activity with zone of inhibition against *Escherichia coli* and *Bacillus subtilis* pathogens*.* Ag–TiO_2_ NCs shows more zone of inhibition as compared to TiO_2_ NPs. In case of Ag–TiO_2_ NCs, release of silver ions from NCs enhances its power to bind with bacterial enzymes which are responsible for inactivating the bio-cells by penetrating the cell walls leading to damage of the bacteria (Gupta et al. 2017).

**Table 1:** Zone of inhibition (mm) at different concentrations of green-synthesised Ag–TiO_2_ NCs and TiO_2_ NPs against Gram-positive and Gram-negative bacteria

Concentration (µg/ml)	Zone of inhibition in mm							
	*E. coli*		*S. aureus*		*B. subtilis*		*P. aeruginosa*	
	TiO_2_	Ag–TiO_2_	TiO_2_	Ag–TiO_2_	TiO_2_	Ag–TiO_2_	TiO_2_	Ag–TiO_2_
25	9.8	11	5	5	7	9	2	2
50	12	13	7	8	10	11	4	4.5
75	16	18	10	13	15	17	7	8
100	19	22	11	14	18	20	9	9.5

**Fig. 7:**
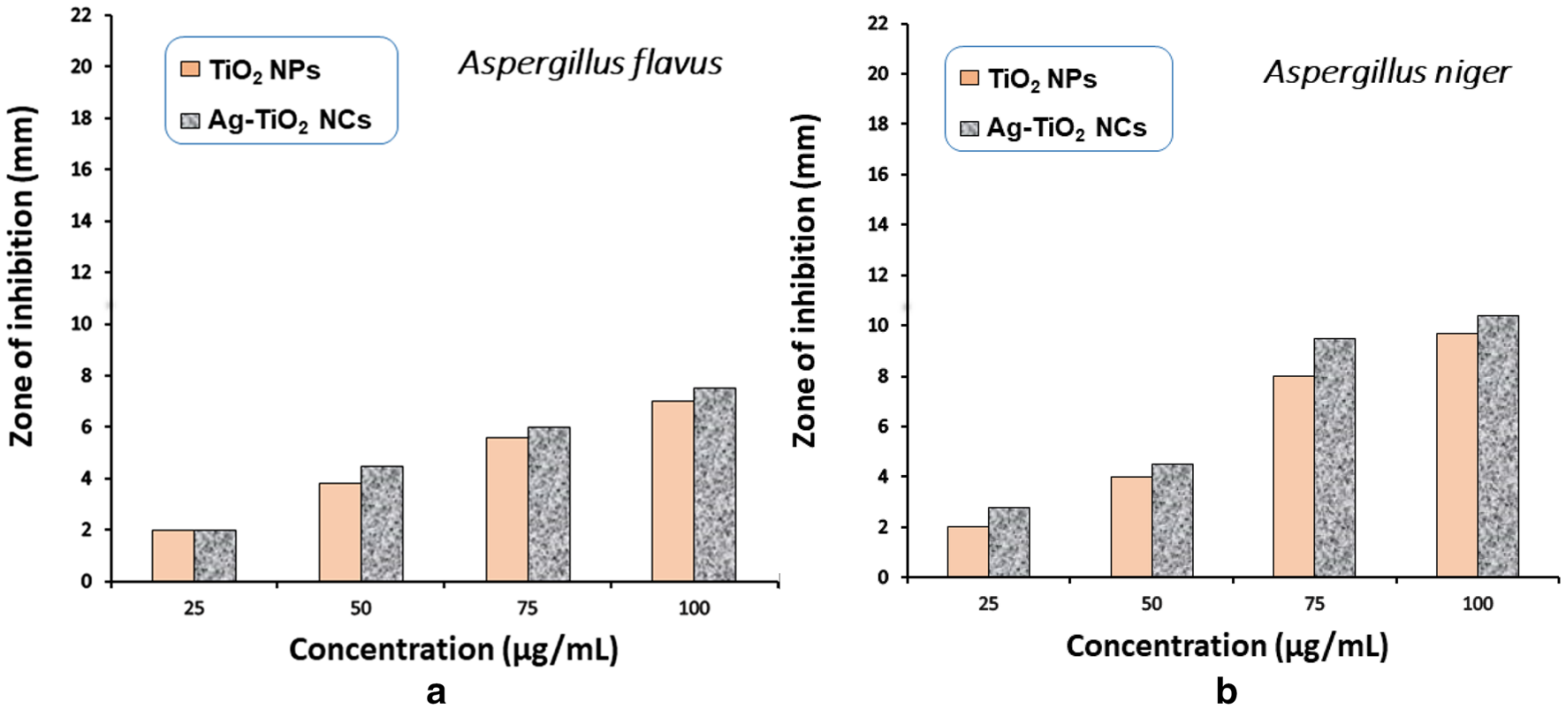
Antifungal activity of green-synthesised Ag–TiO_2_ NCs and TiO_2_ NPs at different concentrations against two fungi **a** *A. flavus* and **b** *A. niger*

Similarly, antifungal activity of green-synthesised Ag–TiO_2_ NCs and TiO_2_ NPs were studied against two selected fungi *Aspergillus niger* and *Aspergillus flavus* illustrated in Fig. 7. Significant antifungal activity of synthesised Ag–TiO_2_ NCs was exhibited against *A. niger* while low activity was observed against *A. flavus*. From the minimum inhibitory concentration (MIC) values, it is clearly seen that considerably low amount of green Ag–TiO_2_ NCs (25 µg/ml) was able to disrupt the fungal cell membrane and lead to its death (Table 2). Similarly as in antibacterial assay, antifungal activity of synthesised Ag–TiO_2_ NCs shows more zone of inhibition as compared to TiO_2_ NPs. The smaller particle size achieved under sonication also contributed the higher antifungal activity of synthesised Ag–TiO_2_ NCs as smaller particle size have large surface to volume ratio due to which more number of drugs molecules get adsorbed on this surface that are expected to be work as a potent agent in disrupting the cell walls (Jalal et al. 2018). The results indicate that synthesised Ag–TiO_2_ NCs are a potent antimicrobial agent carrying more capacity to kill the microbes compared to TiO_2_ NPs.

**Table 2:** Zone of inhibition (mm) at different concentrations of green-synthesised Ag–TiO_2_ NCs and TiO_2_ NPs against two fungi

Concentration (µg/ml)	Zone of inhibition in mm			
	*Aspergillus niger*		*Aspergillus flavus*	
	TiO_2_	Ag–TiO_2_	TiO_2_	Ag–TiO_2_
25	5	7	2	2
50	7	9.5	4	4.5
75	9.8	12	5.8	6
100	10.5	13	7	7.6

However, from a very long time, *Origanum majorana* is a famous herb used in traditional medicines and its remarkable biological activities added advantage along with Ag NPs and TiO_2_ NPs own potent biological properties that would greatly promote green synthesis of Ag–TiO_2_ NCs. Using this popular herb and from previous studies, it was also observed that biosynthesised NPs had showed higher antimicrobial activity than pure chemically synthesized NPs (Santhoshkumar et al. 2014).

### Antioxidant activity of Ag–TiO_2_ NCs

**Fig. 8:**
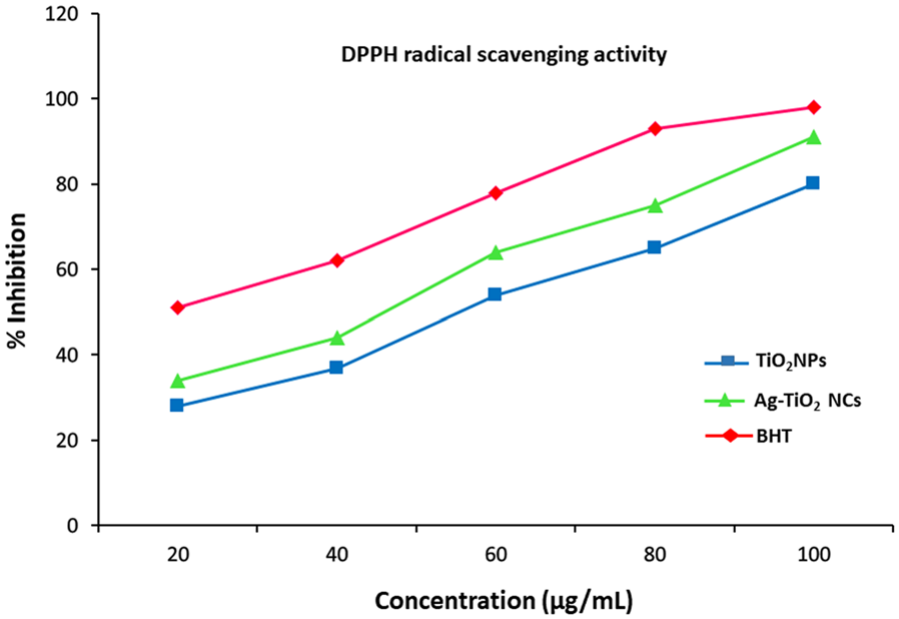
DPPH assay showing enhanced antioxidant activity of green-synthesised Ag–TiO_2_ NCs at different concentrations

An antioxidant compound works in neutralising the free radicals to stop the oxidation process. The antioxidant assay of green-synthesised Ag–TiO_2_ NCs and TiO_2_ NPs were investigated against DPPH at different concentrations (Fig. 8). Significantly, high radical-scavenging activity was observed in Ag–TiO_2_ NCs as compared to TiO_2_ NPs suggesting the presence of silver particles enhances NC antioxidant nature by efficiently separating electron–hole pairs (Hatano et al. 1989). Capped nanocomposites were found to be potent free radical scavenger comparable to standard BHT. The results showed that DPPH free radical scavenging is inhibited by Ag–TiO_2_ NCs in a dose-dependent manner, i.e. with continuous increment in NC concentration, the scavenging activity was also increased.

**Fig. 9:**
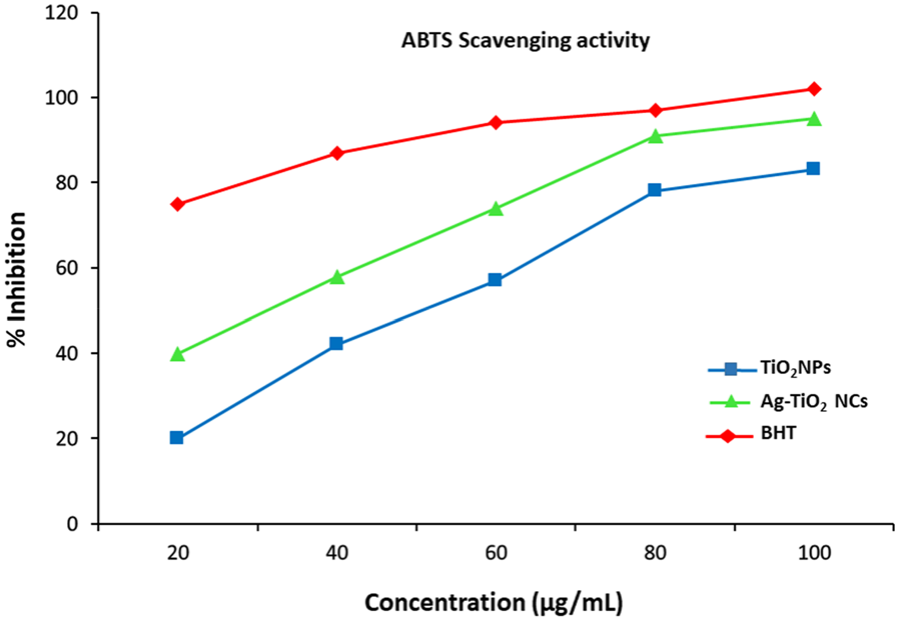
ABTS assay showing enhanced antioxidant activity of green-synthesised Ag–TiO_2_ NCs at different concentrations

The antioxidant assay at different concentrations of green-synthesised Ag–TiO_2_ NCs and TiO_2_ NPs were investigated against ABTS (Fig. 9). Leaves of *Origanum majorana* contain a high content of bioactive compounds (polyphenols and essential fatty acids oils) that have ability for potential scavenging owing to their hydroxyl groups (Kabeer et al. 2017). As similar to DPPH scavenging, ABTS was also found dose-dependent activity. Green-synthesised Ag–TiO_2_ NCs showed strong activity than TiO_2_ NPs via inhibiting ABTS radical and comparable to ascorbic acid (standard reference antioxidant) at higher concentrations.

**Fig. 10:**
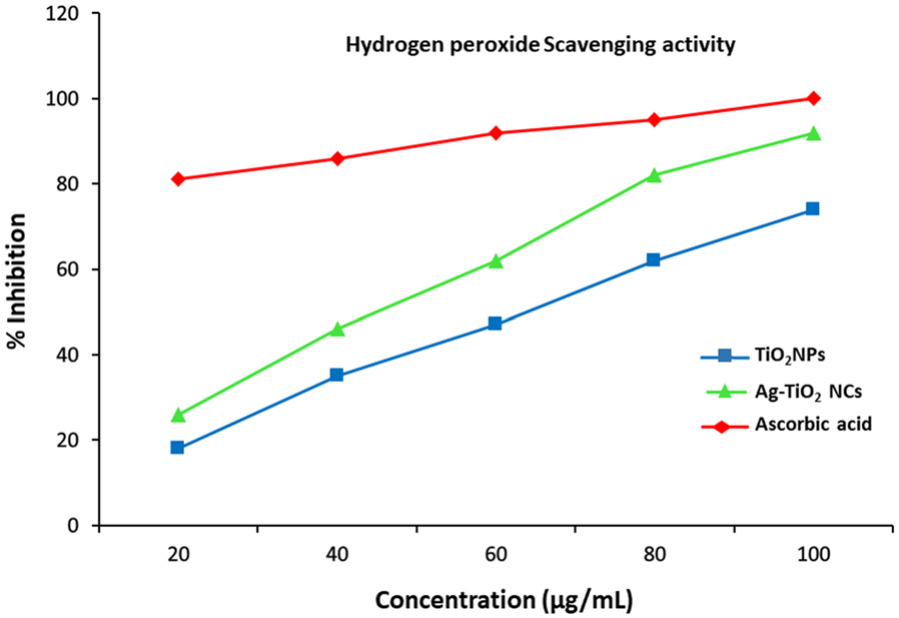
H_2_O_2_ assay showing enhanced antioxidant activity of green-synthesised Ag–TiO_2_ NCs at different concentrations

Green-synthesized Ag–TiO_2_ NCs were capped by the phytoconstituents found in *Origanum majorana* leaf extract that could scavenge a variety of free radicals. The phenolic content present in leaf extract can easily donate electron to hydrogen peroxide and thereby neutralising it into water (Lateef et al. 2017). The hydrogen peroxide-scavenging assay at different concentrations of green-synthesised Ag–TiO_2_ NCs and TiO_2_ NPs were investigated (Fig. 10). Results showed that Ag–TiO_2_ NCs exhibited good-scavenging potential as compare to TiO_2_ NPs. The capped Ag–TiO_2_ NCs showed close scavenging activity to standard ascorbic acid only at high concentration (100 µg/ml) and indicated that H_2_O_2_ activity is also a dose-dependent activity.

Phytoconstituents present in leaf extract of *Origanum majorana* are popular for their antioxidant nature along with Ag and TiO_2_ own antioxidant property which are responsible for further enhancing its scavenging activity and made it more active and potent. From above results, it can be suggested that eco-friendly green Ag–TiO_2_ NCs synthesized using *Origanum majorana* leaf extract under ultrasound irradiation could be a promising candidate for antioxidant drugs more than TiO_2_ NPs and can be a best substitute of chemically synthetic ones.

## Conclusion

In this study, Ag–TiO_2_ NCs were synthesised via a simple, cost-effective, eco-friendly approach using leaf extract of *Origanum majorana* as a bio-reductant under ultrasound irradiation for the first time. The aqueous leaf extract containing phytoconstituents was used for reduction and stabilisation of NCs. The synthesised Ag–TiO_2_ NCs were characterized by UV–Vis, FTIR, XRD, SEM–EDS and TEM analysis. Antimicrobial and antioxidant activities of Ag–TiO_2_ NCs were performed and found with dose-dependent variation. Green-synthesised Ag–TiO_2_ NCs showed excellent biological activities compared to TiO_2_ NPs and close to standard. There are many advantages of applying the present sonochemical route for synthesis such as low temperature, short duration of time, and well dispersibility of doped metal on the surface providing smaller size particles with high yields. All data analysed during this study are included in this article.

## Data Availability

All data analysed during this study are included in this article.

## Abbreviations

ABTS: 2,2′-Azino-bis(3-ethylbenzthiazoline-6-sulfonic acid
ATP: Adenosine triphosphate
BHT: Butylated hydroxyltoluene
DPPH: 1,1-Diphenyl-2-picrylhydrazyl
EDS: Energy-dispersive X-ray spectroscopy
FCC: Face-centred cubic
FTIR: Fourier transform infrared
LSPR: Localised surface plasmon resonance
MIC: Minimum inhibitory concentration
NCs: Nanocomposites
NPs: Nanoparticles
ROS: Reactive oxygen species
SEM: Scanning electron microscopy
TEM: Transmission electron microscopy
TTIP: Titanium (IV) isopropoxide
UV–Vis: UltraViolet–Visible
XRD: X-ray diffraction
